# Bi-Functional Peptides as a New Therapeutic Tool for Hepatocellular Carcinoma

**DOI:** 10.3390/pharmaceutics13101631

**Published:** 2021-10-06

**Authors:** Eric Savier, Lorena Simon-Gracia, Frederic Charlotte, Pierre Tuffery, Tambet Teesalu, Olivier Scatton, Angelita Rebollo

**Affiliations:** 1 Department of Hepatobiliary and Liver Transplantation Surgery, AP-HP, Pitié–Salpêtrière Hospital, Sorbonne Université, 75006 Paris, France; eric.savier@aphp.fr (E.S.); olivier.scatton@gmail.com (O.S.); 2 Sant Antoine Research Center (CRSA), Institut Nationale de la Santé et la Recherche Médicale (Inserm), Institute of Cardiometabolism and Nutrition (ICAN), Sorbonne Université, 75006 Paris, France; 3 Laboratory of Precision and Nanomedicine, Institute of Biomedicine and Translational Medicine, University of Tartu, 50090 Tartu, Estonia; lorenasimongracia@gmail.com (L.S.-G.); tambet.teesalu@ut.ee (T.T.); 4 Department of Pathology, AP-HP, Pitié–Salpêtrière Hospital, 75006 Paris, France; frederic.charlotte@aphp.fr; 5 Biologie Fontionelle Adaptative (BFA), Unité Mixte de Recherche (UMR) 8251, Centre National de la Recherche Scientifique (CNRS) ERL U1133, Inserm, Université de Paris, 75006 Paris, France; pierre.tuffery@univ-paris-diderot.fr; 6 Center for Nanomedicine and Department of Cell, Molecular and Developmental Biology, University of California, Santa Barbara, CA 93106, USA; 7 Faculté de Pharmacie, Unité des Technologies Chimiques et Biologiques pour la Santé (UTCBS), Inserm U1267, Centre National de la Recherche Scientifique CNRS UMR8258, Université de Paris, 75006 Paris, France

**Keywords:** hepatocellular carcinoma, tumor-penetrating peptides, interfering peptides

## Abstract

Background: The interfering peptides that block protein–protein interactions have been receiving increasing attention as potential therapeutic tools. Methods: We measured the internalization and biological effect of four bi-functional tumor-penetrating and interfering peptides into primary hepatocytes isolated from three non-malignant and 11 hepatocellular carcinomas. Results: These peptides are internalized in malignant hepatocytes but not in non-malignant cells. Furthermore, the degree of peptide internalization correlated with receptor expression level and tumor aggressiveness levels. Importantly, penetration of the peptides iRGD-IP, LinTT1-IP, TT1-IP, and RPARPAR-IP induced apoptosis of the malignant hepatocytes without effect on non-malignant cells. Conclusion: Receptor expression levels correlated with the level of peptide internalization and aggressiveness of the tumor. This study highlights the potential to exploit the expression of tumor-penetrating peptide receptors as a predictive marker of liver tumor aggressiveness. These bi-functional peptides could be developed for personalized tumor treatment.

## 1. Introduction

Despite significant progress in translational cancer research, advances in the design of targeted anti-cancer therapies have remained disappointingly slow [1,2,3,4,5,6]. The two most important issues with current cancer therapies are the lack of tumoral specificity and the lack of selectivity. Treatments thus induce off-target effects and adverse side effects, and the amount of drug that actually reaches its target remains relatively low. Consequently, there is a real need for selective anti-cancer drugs.

Various targeted delivery strategies have been developed in an effort to overcome these limitations. One strategy is the use of Tumor-Penetrating Peptides (TPP), which are recognized as tumor-specific drug delivery vehicles that can penetrate into tumor cells to deliver cargo. TPPs are internalized via specific receptors expressed on tumor cells and vasculature [7,8], and are characterized by the presence of the C-end Rule (CendR) motif with the consensus sequence R/KXXR/K [9,10]. This motif has to be C-terminally exposed to allow tumor-specific binding and penetration via the Neuropilin-1 (NRP-1) receptor.

RPARPAR is a prototypic CendR peptide that binds and internalizes via the NRP-1 receptor [11,12]. Another widely used TPP is iRGD (CRGDKGPDC), which is recruited to tumors via interaction with the integrins αvβ3/5 through the RGD motif. On the cell surface, the tumor proteases cleave iRGD to C-terminally expose the CRGDK CendR motif that triggers internalization with the NRP-1 receptor [13]. The cyclic TT1 and its linear version, LinTT1, bind first to the p32 protein expressed on the tumor cell surface [14]. Both are also cleaved by tumoral proteases, thus exposing the CendR sequence that can then bind to NRP-1 [14,15,16,17,18,19].

TPPs are widely used as tumor-homing affinity ligands in targeted therapies, as they combine tumor specificity with cargo to reduce toxicity and increase efficacy [17,20,21,22,23,24,25]. In previous work, we paired TPPs with an Interfering Peptide (IP) that blocks the interaction between the phosphatase PP2A and its physiological inhibitor, the oncoprotein SET. This leads to bi-functional peptides that are able to specifically target tumoral cells where, once internalized, they dissociate the PP2A/SET interaction [26].

Hepatocellular Carcinoma (HCC) is a primary liver cancer that originates from hepatocytes [27]. HCC is the sixth most frequent cancer and the fourth leading cause of cancer-related mortality worldwide. Risk factors for HCC include viral infection, alcohol abuse, non-alcoholic fatty liver disease, certain toxins, and genetic diseases. These factors are responsible for chronic liver inflammation, fibrosis, and ultimately cellular transformation and liver function impairment [28]. Multimodal lines of therapy against HCC include surgical resection, chemotherapy, or radiotherapy. Tyrosine kinase inhibitors have some efficacy but are contraindicated in cases that involve altered liver function. Immunotherapy treatments hold promise for treating HCC [28], but more efficient targeted therapies are needed. An alternative way to treat HCC could be to use a dual-peptide strategy combining a TPP with an IP. Here, we show that the fused TPP-IPs can selectively internalize into primary tumoral hepatocytes isolated from HCC patients. Our results show that level of TPP-IP receptor expression by HCC tumor cells correlates with degree of peptide internalization and tumor aggressiveness, which raises prospects for a selective liver tumor-targeting approach.

## 2. Materials and Methods

### 2.1. Patients

Samples of benign and tumoral liver were collected from 14 patients. All patients gave informed consent. Samples 1 to 3 correspond to non-malignant tumors or necrotic HCC (following sorafenib treatment), and samples 4 to 14 correspond to HCC (Table 1). A tumor aggressiveness score was calculated based on histological or biological factors known to be associated with poor prognosis. This tumor aggressiveness score included tumor encapsulation [29], tumor differentiation [30], presence of satellite nodules, vascular invasion, macrotrabecular type [31], and log_10_ of the preoperative Alpha Feto Protein (AFP) value [32]. Tumors that scored 0 had zero aggressiveness, tumors that scored <6 were considered moderately aggressive, and tumors that scored >6 were considered highly aggressive.

### 2.2. Peptide Synthesis and Sequences

The peptides were synthesized in an automated multiple peptide synthesizer with solid-phase and standard Fmoc chemistry (GL Biochem, Shanghai, China). The characterization was performed by High-Performance Liquid Chromatography (HPLC, Shimadzu France, Marne-la-Valle) and Mass Spectrometry (MS, Bruker, Wissembourg, France). For internalization experiments, the peptides were synthesized with a fluorochrome (FITC, Sigma-Aldrich, Saint Quentin, France). The peptides and sequences used are shown in Table 2.

### 2.3. Isolation and Culture of Primary Human Tumoral Hepatocytes

Healthy hepatocytes were isolated from patient samples following a protocol previously described [26]. Human tumoral hepatocytes were isolated from tumoral liver samples collected from adult patients undergoing surgery. Samples were cut into small pieces and treated with 4 mL of dispase (Gibco, Ref 17105-041, Thermo Fisher, France; 10 mg/mL in PSA buffer) (NaCl 8 g/L, KCl 0.2 g/L, glucose 1 g/L, NaHCO_3_ 0.35 g/L, phenol red 1 mL/L; Thermo Fisher, France) and 2 mL of collagenase type I (Gibco Ref 17100-017; 5 mg/mL in PSA buffer). Samples were incubated at 37 °C under agitation for a maximum of 1 h, and then the solution was filtered and passed through needles of different diameters. The volume was filled up to 50 mL with culture medium and centrifuged at 177· *g* for 5 min. Arginase expression confirmed that the isolated cells were hepatocytes. The supernatant was discarded, and the cells were cultured in DMEM medium (Thermo Fisher, France) supplemented with 10% Fetal Calf Serum (FCS, Gibco, Thermo Fisher, France) and antibiotics until treatment with peptides. The hepatocytes were maintained in culture for no more than 36 h to ensure that they did not enter differentiation.

### 2.4. Quantification of Cellular Internalization

Primary human hepatocytes were seeded overnight on 24-well plates and then incubated for 4 h with FITC-labeled peptides. After treatment, cells were detached, treated with trypsin (Gibco, Thermo Fisher, France) to remove non-internalized peptides, washed twice with PBS (Gibco, Thermo Fisher, France) to remove free peptides, and resuspended in 200 μL of PBS. FITC fluorescence intensity of internalized peptides was measured using a FACSCanto II flow cytometry system (Beckton Dickinson, Franklin Lakes, NJ, USA). Data were analyzed using FACSDiva 6.1.3 software (DB Biosciences, Franklin Lakes, NJ, USA). Healthy primary hepatocytes were used as control. For detection of TPP receptors on the cell surface, anti-p32 (Sigma Aldrich, St. Louis, MO, USA, AB2991), anti-NRP-1 (antibody generated in house, prepared by immunizing rabbits with human recombinant NRP1, followed by affinity purification) [24], and anti-integrin v/β3 (Abcam, Cambridge, UK, ab203123) antibodies were incubated with the cells for 30 min at room temperature. Cells were then washed and incubated with fluorophore-labeled secondary goat anti-mouse antibody (Alexa Fluor 647 goat anti-mouse, Thermo Fisher, Waltham, MA, USA, A-21238) or goat anti-rabbit antibody (Thermo Fisher, Waltham, MA, USA, A48285) for 10 min. Cells were then washed again, and receptor expression was analyzed by flow cytometry as described above.

### 2.5. Immunohistochemistry

The immunostaining procedure was performed on formalin-fixed, deparaffinized, 3µm-thick sections using a Ventana Benchmark Ultra platform (Roche Diagnostics, Basel, Switzerland) and the Ultraview visualization system (Roche Diagnostics, Basel, Switzerland) according to the manufacturer′s instructions. The following primary antibodies were used: mouse monoclonal anti-CK19 antibody (dilution 1/100; clone RCK108; ref. M088801-2, Agilent, Santa Clara, CA, USA) followed by CC1 antigen retrieval buffer (36 min, 95 °C) and an antibody incubation time of 20 min at 20 °C; mouse monoclonal anti-human hepatocyte (HepPar) (dilution 3/100; clone OCH1E5; ref. M715801-2, Agilent, Santa Clara, CA, USA) followed by CC1 antigen retrieval buffer (64 min, 95 °C) and an antibody incubation time of 32 min at 20 °C; mouse monoclonal anti-human Glypican-3 (prediluted; clone 1G12; ref. F/261M-98, MM, Brignais, France) followed by CC1 antigen retrieval buffer (64 min, 95 °C) and an antibody incubation time of 32 min at 37 °C; mouse monoclonal anti-human b-catenin (prediluted; clone 14; ref. 05269016001, Roche Diagnostics, Basel, Switzerland) followed by CC1 antigen retrieval buffer (64 min, 95 °C) and an antibody incubation time of 32 min at 37 °C, and mouse monoclonal anti-human glutamine synthetase (prediluted; clone GS6; ref. 07107757001, Roche Diagnostics, Basel, Switzerland) followed by antigen retrieval protease (4 min, 20 °C) and an antibody incubation time of 40 min at 20 °C.

### 2.6. Detection of Apoptosis by Annexin-V Staining

The degree of apoptosis induced by the four TPP-IPs on primary benign and tumoral treated hepatocytes was measured by flow cytometry on cells stained with annexin-V FITC (Biosciences, Fischer Scientific, Hampton, NH, USA). The primary cells were incubated with the peptides for 12 h at 37 °C in DMEM supplemented with 10% FCS (Gibco, Thermo Fisher, France), then washed and treated according to the manufacturer’s protocol. Level of apoptosis was measured using FACSCanto II flow cytometry system (Becton Dickinson Biosciences, Franklin Lakes, NJ, USA).

### 2.7. Immunoprecipitation and Western Blotting

MDA-MB231 cells (ATCC, HTB-26) (5 × 10^6^) were lysed for 20 min at 4 °C in lysis buffer (50 mM Tris pH8, 1% NP40, 137 mM NaCl, 1 mM MgCl_2_, 1 mM CaCl_2_, 10% glycerol and protease inhibitor mixture, Sigma Aldrich, St. Louis, MO, USA). Lysates (500 μg) were immunoprecipitated with the appropriate antibody overnight at 4 °C, and protein A/G Sepharose (Santa Cruz, Dallas, TX, USA) was added for 1 h at 4 °C. After washing with TBST (20 mM Tris-HCl pH7.5, 150 mM NaCl, 0.05% Tween 20; Gibco, Thermo Fisher, France), the PP2A/SET interaction was competed using 1 mM of PP2A/SET or Ras/Raf IP (GL Biochem, Shanghai, China) for 30 min at room temperature. After several washing steps, immunoprecipitates were separated by SDS-PAGE, transferred to nitrocellulose, and blotted with anti-PP2A antibody (Sigma Aldrich, St. Louis, MO, USA). The membrane was washed and incubated with HRP-conjugated secondary antibody (Dako, Hamburg, Germany, 1:1000 dilution). Protein detection was performed using the ECL system (Bio-Rad, Hercules, CA, USA). The blot was also hybridized with anti-SET antibody as internal control (Thermo Fischer, Waltham, MA, USA, MA5-34662).

### 2.8. Statistical Analysis

The data were analyzed using SigmaPlot version 12.0, Systat Software, Inc. (D-40699, Erkrath, Germany) and logarithmic regression with StatView version 5.0 for Windows SAS Institute Inc. Statistical tests used include *t*-tests and a Mann-Whitney rank sum test or Pearson correlations, as appropriate. Values of *p* < 0.05 were considered statistically significant.

## 3. Results

### 3.1. Clinical Characteristics of the Patients and Tumor Aggressiveness Classification

Samples from 14 patients were analyzed. Of these, three were from non-malignant tumors and 11 from HCC. The patient population had a median age of 62 years (range: 47–78 years) with a large predominance of males (71%). Clinical aggressiveness was calculated according to six parameters: AFP, non-encapsulation, satellite nodules, vascular embolization, differentiation, and macrotrabecular type (Table 1). Samples 1 to 3 corresponded to zero aggressiveness hepatocellular adenoma, necrotic tissue and angiomyolipoma, respectively. Samples 4 to 14 corresponded to HCC of moderate or high aggressiveness and were classified using the following parameters: for encapsulation, non-encapsulated = 0, partially encapsulated = 1; for differentiation, well-differentiated = 1, moderately differentiated = 2, undifferentiated = 3; for satellite nodes, positive = 1, negative = 0; for vascular invasion, positive = 1, negative = 0; for macrotrabecular type, positive = 1, negative = 0.

### 3.2. Immunohistochemical Characteristics of the Patients

The immunohistochemical markers that were analyzed in the patient samples were: CK19, to differentiate HCC from cholangiocarcinoma; HepPar, a marker that differentiates HCC from metastatic carcinoma [33]; GPC3, a member of the glypican family involved in progression of HCC [34]; β-catenin, a marker of development and progression of HCC [35]; and glutamine synthetase [36], which may enhance metastatic potential in HCC. Absence of CK19 expression confirmed that the patient samples corresponded to HCC but not to cholangiocarcinoma. Note that samples from patients #7, #8, #11 and #12, which were classified as highly aggressive HCC, showed the highest levels of HepPar marker expression. Similarly, samples from patients #6 and #7, which were also classified as highly aggressive HCCs, expressed the highest levels of glutamine synthase (Table 3).

### 3.3. In Vitro Competition against PP2A/SET Interaction

**Figure 1:**
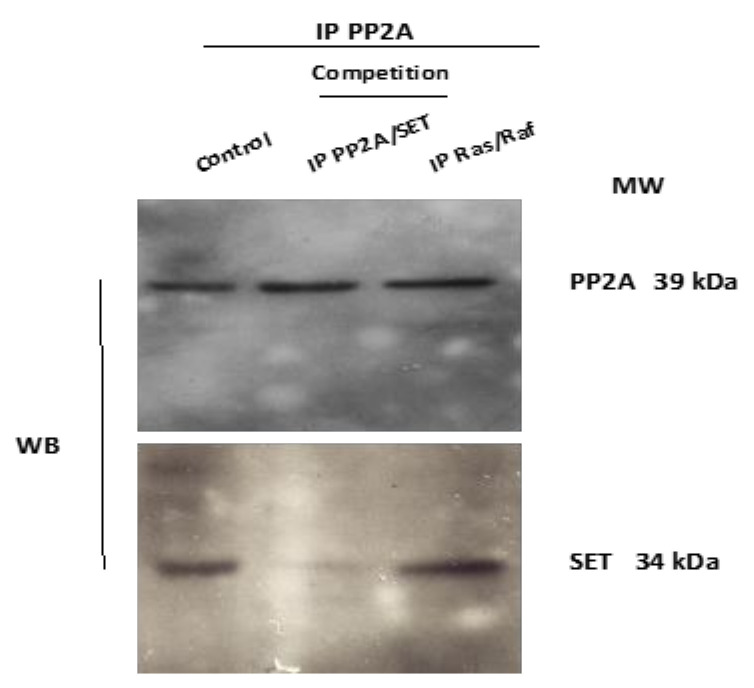
The IP disrupted PP2A/SET interaction in vitro. Lysates were immunoprecipitated with anti PP2A antibody. The PP2A/SET interaction was competed in vitro with 1 mM of the PP2A/SET IP and with an irrelevant Ras/Raf IP used as a negative control. The total amount of PP2A was used as internal control.

In vitro competition testing was performed to confirm that the IP targeted the PP2A/SET interaction. Lysates from MDA-MB231 cells were immunoprecipitated with anti-PP2A antibody, and the interaction with SET was competed using IP PP2A/SET (Figure 1). SET was detected in the control immunoprecipitates and in immunoprecipitates after competition with the IP disrupting the Ras/Raf interaction (peptide sequence: MEHIQGAWKTISGFGLK), whereas the levels detected were much lower after competition with 1 mM of the IP blocking the PP2A/SET interaction. PP2A was used as internal control of protein loading.

### 3.4. Internalization of Tumor-Penetrating and Interfering Peptides (TPP-IP) into Primary Tumoral Hepatocytes via Specific Receptors

We generated four bi-functional peptides composed of a TPP (iRGD, RPARPAR, LinTT1, or TT1) paired with the IP blocking the interaction between the phosphatase PP2A and its physiological inhibitor, the oncoprotein SET. These peptides penetrated specifically into tumoral B-cells [26]. We analyzed the intracellular penetration of these TPP-IP in a group of 14 samples of non-malignant tumors (samples #1 to #3) or HCC (samples #4 to #14), graded according to histological tumor type.

**Figure 2:**
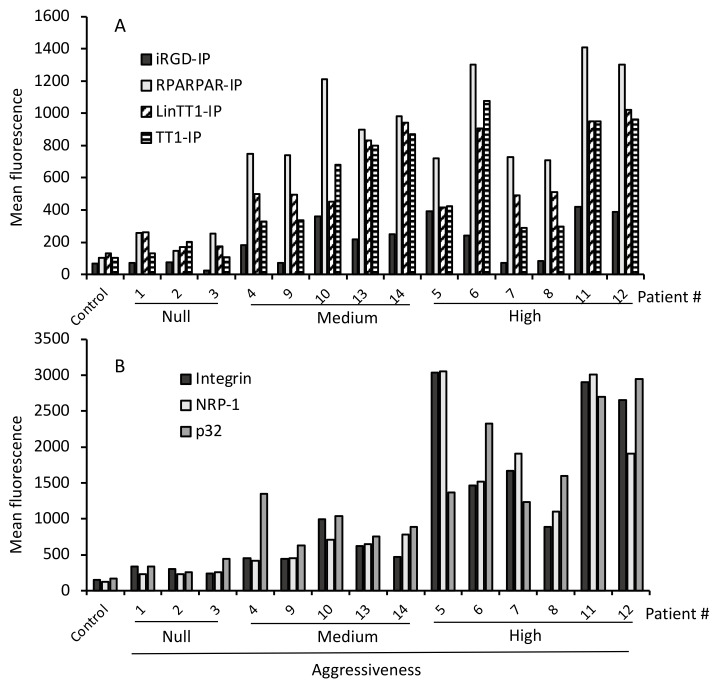
Selective internalization of TPP-IPs and receptor expression in malignant hepatocytes. (**A**) TPP-IP internalization. Hepatocytes isolated from benign or malignant liver patient samples were incubated for 4 h with 25 μM of FITC-labeled peptides. The mean fluorescence of internalized peptides was analyzed by flow cytometry and compared to control healthy hepatocytes. Internalization of the TPP-IPs was significantly higher in HCC compared to non-malignant tumors (HCC versus non-malignant tumors or normal hepatocytes respectively, *n* = 4 to 11 per group, mean ± standard error, iRGD-IP: 61 ± 12 vs. 244 ± 40, *p* = 0.022; RPARPAR-IP: 191 ± 38 vs. 977 ± 83, *p* = 0.005; LinTT1-IP: 184 ± 27 vs. 682 ± 73, *p* = 0.002; TT1-IP: 137 ± 23 vs. 637 ± 93, *p* = 0.005). (**B**) Receptor expression. Hepatocytes isolated from non-malignant or tumoral liver samples were incubated with antibodies against NRP-1, p32, and integrin v/b3, followed by an APC-labeled secondary antibody. Samples were analyzed by flow cytometry. Healthy hepatocytes were used as control. Receptor expression levels were significantly higher in tumoral hepatocytes than in non-malignant tumors (HCCs vs. non-malignant tumors or normal hepatocytes, respectively, *n* = 4 to 11 per group, Integrin v/b3: 254 ± 40 vs. 1416 ± 306, *p* = 0.005; NRP-1: 211 ± 31 vs. 1410 ± 290, *p* = 0.005; p32: 303 ± 58 vs. 1530 ± 238, *p* = 0.01).

Figure 2A shows that none of the four TPP-IPs penetrated non-malignant tumors. In HCC (samples #4 to #14), iRGD-IP showed the lowest level of internalization but with a significant difference between benign and aggressive tumors (*p* = 0.02). Bi-functional peptides RPARPAR-IP (*p* = 0.005), LinTT1-IP (*p* = 0.002), and TT1-IP (*p* = 0.005) showed higher levels of penetration in tumoral hepatocytes, again with a significant difference compared to non-malignant samples (Figure 2A). Interestingly, RPARPAR-IP showed the highest level of internalization in HCC samples, ahead of LinTT1-IP and TT1-IPs which showed very similar levels of internalization (Figure 2A). Crucially, none of the TPP-IPs internalized into healthy hepatocytes (control in Figure 2A).

Given that these peptides are internalized by tumoral hepatocytes via specific receptors on tumoral cells, we analyzed the expression of integrin v/β3, p32 and NRP-1. Figure 2B shows that samples #1 to #3 (non-malignant tumors) and healthy control hepatocytes all showed very low levels of cell surface receptor expression, whereas samples #4 to #14 (HCC tumors) showed significantly higher receptor expression levels compared to non-malignant tumors (*p* = 0.05 for integrin v/β3; *p* = 0.05 for p32; *p* = 0.05 for NRP-1). We previously showed that the IP without TPP failed to internalize into malignant B cells and tumoral hepatocytes, whereas a non-tumoral-specific cell-penetrating peptide alone or combined with the IP effectively internalized in both malignant and healthy B cells and hepatocytes. The new results reported here confirm that the specific internalization of the TPP-IPs into tumor cells is due to internalization via specific receptors.

### 3.5. TPP Internalization and Receptor Expression Correlated with Tumor Aggressiveness

iRGD is recruited via interaction with integrins and then cleaved by tumoral proteases, thus allowing interaction with the NRP-1 receptor. Similarly, LinTT1 and TT1 first bind to p32, a mitochondrial protein aberrantly expressed on the cell surface of tumoral cells and are then cleaved by proteases expressed by the tumor cells, allowing them to interact with NRP-1. Finally, the RPARPAR peptide binds directly to tumoral cells expressing NRP-1.

**Figure 3:**
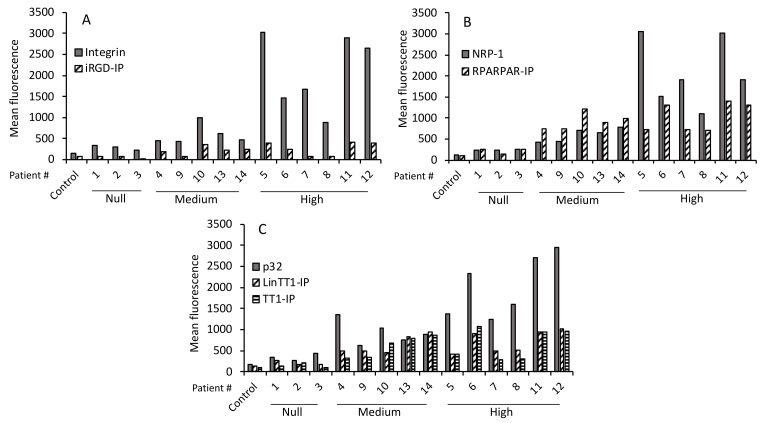
Primary receptor expression and internalization of TPP-IPs. (**A**) Expression of primary receptor integrin and iRGD-IP internalization. (**B**) Expression of NRP-1 receptor and RPARPAR-IP internalization. (**C**) Expression of the primary p32 receptor and LinTT-1-IP and TT1-IP internalization. Data are from Figure 2.

We tested whether there was a correlation between the level of primary receptor expression on the tumoral cells and level of peptide internalization. Figure 3A shows a low level of iRGD-IP internalization (compared with RPARPAR-IP, LinTT1-IP, and TT1-IP) and variable expression of its receptor, integrin v/β3. The highest levels of integrin v/β3 expression were found in samples from patients #5, #6, #7, #11, and #12, which matched to the samples with the high tumor aggressiveness scores. Moreover, samples #5, #11, and #12 showed a higher degree of internalized iRGD-IP, which also matched with high tumor aggressiveness. A similar pattern was found for NRP-1 receptor expression and RPARPAR-IP internalization (Figure 3B), where the highest level of NRP-1 expression was found in samples from patients #5, #6, #7, #11, and #12, and the highest RPARPAR-IP internalization was found in samples #6, #11, and #12 that also corresponded to the most aggressive tumors. Finally, there was a different pattern of p32 receptor expression and LinTT1-IP/TT1-IP internalization, with the highest expression of the receptor in samples from patients #6, #11, and #12 that were classified as aggressive tumors, and these same samples also showed the highest peptide internalization (Figure 3C).

**Figure 4:**
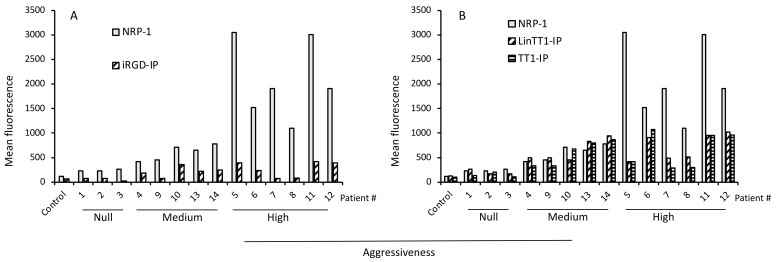
Secondary receptor expression and internalization of TPP-IPs. (**A**) Expression of secondary receptor (NRP-1) and iRGD-IP internalization. (**B**) Expression of secondary receptor (NRP-1) and internalization of LinTT1-IP and TT1-IP. Data are from Figure 2.

Analysis of NRP-1 expression levels in comparison to internalization of iRGD-IP (Figure 4A), LinTT1-IP and TT1-IP (Figure 4B), or RPARPAR-IP (Figure 3B) found that samples with the highest receptor expression also had high tumor aggressiveness scores and showed prominent TPP-IP internalization.

**Figure 5:**
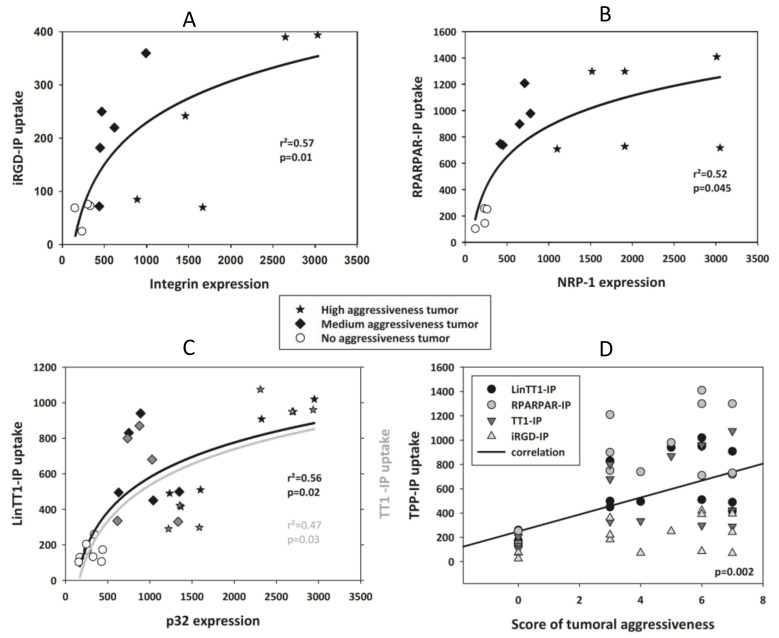
Correlation between receptor expression, peptide internalization, and aggressiveness of HCC. Correlation between integrin expression and internalization of iRGD-IP followed the equation: y = b0 + b1 × log(x). White circle: non-malignant tumor or normal hepatocytes; black squares: samples from medium aggressiveness tumors; black stars: samples from high aggressiveness tumors. (**A**) Integrin expression and iRGD-IP internalization, (b0 = −610 ± 207, b1 = 121 ± 31) (**B**); NRP-1 expression and RPARPAR-IP internalization, (b0 = −1344 ± 600, b1 = 322 ± 89) (**C**), p32 expression and internalization of LinTT1-IP (b0 = −1456 ± 529, b1 = 294 ± 76) and TT1-IP (b0 = −1636 ± 665, b1 = 313 ± 96) (**D**) Correlation between score of aggressiveness and TTP-IP internalization (r = 0.479, standard error of estimate = 333.2, f = y0 + a × x with y0 = 249 ± 83, a = 69.7 ± 17).

Figure 5A–C shows that there was a significant correlation between the levels of primary receptor expression (integrin vβ3, NRP-1 and p32) and peptide internalization (*p* = 0.010 for iRGD-IP; *p* = 0.045 for RPARPAR-IP; *p* = 0.02 for LinTT1-IP; *p* = 0.03 for TT1-IP). Figure 5C also shows that tumor aggressiveness score correlates with TPP-IP internalization (*p* = 0.02).

### 3.6. Apoptotic Effect of TPP-IPs on Tumoral Hepatocytes

**Figure 6:**
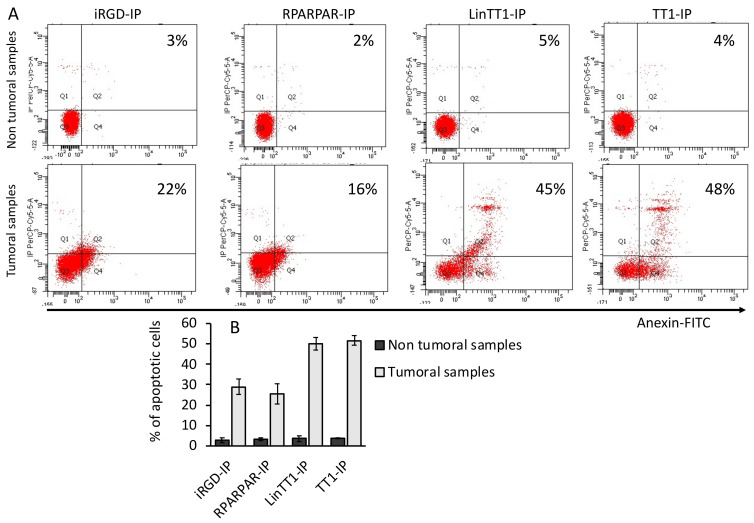
TPP-IPs induce apoptosis in HCC. Hepatocytes isolated from HCC (patient 7) or non-malignant tumors (patient 1) were cultured 12 h with 25 μM and the resulting apoptosis was estimated by annexin-V-FITC staining using flow cytometry. (**A**) Flow cytometry plots from one non-tumoral (patient 1) and one tumoral sample (patient 7). (**B**) Quantification of the percentage of apoptotic cells from three non-tumoral and three tumoral samples. Error bars = ± standard error.

We have previously demonstrated that TPP-IPs induced apoptosis in tumoral B-cells [26]. Figure 6 shows that iRGD-IP, RPARPAR-IP, LinTT1-IP, and TT1-IP induced apoptosis in HCC (sample #7 here) but not in non-malignant samples (sample #1). Apoptotic effect was stronger for LinTT1-IP and TT1-IP peptides, suggesting a tumor-specific induction of apoptosis. 

## 4. Discussion

Liver cancer remains a global health challenge, and its incidence is growing worldwide. It is estimated that by 2025, liver cancer will affect one million people annually [37,38]. The most common form of liver cancer is hepatocellular carcinoma (HCC), which accounts for ~90% of cases. Approximately 25% of HCC tumors present mutations, but they remain undruggable [39,40]. The histology-based definition of the morphological heterogeneity of liver cancer has been modified in an effort to employ personalized therapies for patient treatment [27].

The type of HCC treatment depends on tumor stage, patient performance, and the hepatic functional reserve. The pathogenesis of HCC is a complex multistage process, where angiogenesis plays an important role. For patients with advanced disease, only a handful of kinase inhibitors are approved for therapy, such as cabozantinib, regorafenib, lenvatinib, or sorafenib [41,42,43,44,45]. Anti-angiogenic agents, as well as some monoclonal antibodies, are also approved for use in HCC treatment.

Several therapeutic approaches to specifically target tumoral cells have been investigated. Interfering peptides are emerging as promising therapeutic agents that block intracellular protein–protein interactions [46,47]. The serine/threonine phosphatase PP2A is frequently altered in cancer, either in terms of expression levels or activation [48,49,50]. The physiological inhibitor of PP2A, i.e., the oncoprotein SET, engages with the catalytic subunit of PP2A to block its activation. Competitive interfering peptides able to block the PP2A/SET interaction can therefore restore PP2A activity [51,52]. We have generated four TPP-IPs able to specifically penetrate tumoral hepatocytes and B-cells and induce apoptosis of malignant cells [26]. Here, the four peptides (iRGD-IP, RPARPAR-IP, LinTT1-IP, and TT1-IP) that block the PP2A/SET interaction were able to penetrate tumoral hepatocytes isolated from HCC, but crucially, they were not internalized by non-malignant tumors.

Different parameters have been used to define an aggressiveness score. Recent publications define the aggressiveness score based on four clinical parameters, i.e., tumor size, multifocality, presence of portal vein thrombus, and blood alpha-fetoprotein levels [53,54]. Here we defined an aggressiveness score based on the six parameters indicated in Table 1. Given these criteria, we classified the patients into three groups: non-aggressive (non-malignant tumors), moderately aggressive (score up to five), and highly aggressive (score of six and higher). The results show that there was a correlation between the number of TPP receptors expressed by tumoral cells, level of TPP-IP internalization, and HCC aggressiveness.

Treatment of HCC cells with the bi-functional peptides tested here led to a higher level of apoptosis in HCC cells than in non-malignant samples. The penetration-induced apoptosis was mediated by the associated IP and the specific tumoral penetration. Internalization of the TPP-IPs is the result of a multistep mechanism. First, the bi-functional peptides are associated to their primary receptors (αvβ3/5 integrins for iRGD, p32 for LinTT1, and TT1and NRP-1 for RPARPAR). After proteolytic cleavage by tumoral proteases, they bind to the NRP-1 receptor, triggering cellular internalization. One possible explanation for the lower internalization of iRGD-IP, TT1-IP, and LinTT1-IP compared to RPARPAR-IP could be that after the incubation times used here, only a fraction of these peptides gets cleaved to expose the CendR motif. The involvement of several tumor-dependent steps renders this mechanism highly selective toward tumor cells expressing integrin, p32, and NRP-1 receptors [7,10,14,15,16]. There are several lines of evidence showing that NRP-1 mediates angiogenesis and that increased NRP-1 expression correlates with a decrease in tumor progression, angiogenesis, and immune evasion [55,56]. Overexpression of NRP-1 in vitro and in vivo correlates with decreased tumor vascularization and apoptosis, suggesting a direct correlation between level of NRP-1 expression and aggressiveness of the tumor [55,56]. Similarly, the level of p32 expression by tumoral cells and tissues has been associated with cancer progression and metastasis in several cancers, such as thyroid, pancreatic, gastric, and lung cancer [57].

Uncontrolled tumor cell proliferation and escape from apoptosis play an important role in HCC growth, which makes inhibition of cancer proliferation and induction of apoptosis a crucial target for HCC treatment. Patients with late-stage HCC currently have to rely on systemic chemotherapy [58]. However, the prognosis of patients undergoing chemotherapy for HCC is severely compromised by the toxic side effects of the drugs and by the emergence of drug-resistant HCC tumors [59]. Consequently, there is a real need to search for new targets to treat liver tumors.

Protein phosphatase PP2A and its physiological inhibitor are implicated in HCC as well as other types of cancers. PP2A is a tumor suppressor that negatively regulates many signaling pathways associated with cancer progression by dephosphorylating crucial proteins in these pathways, such as Wnt/β-catenin, PI3K, MAPK, and so on [60,61].

PP2A phosphatase inhibitors have been shown to have therapeutic effects against HCC in clinical trials [62,63], suggesting that PP2A is a promising target for HCC treatment. Unfortunately, the extensive constitutive expression of PP2A in normal tissues, as well as the many PP2A partners and signaling pathways in which PP2A is involved, have bottlenecked the efforts to exploit PP2A as a target for therapeutic intervention. In addition, in clinical trials, phosphatase inhibitors exert a toxic effect against normal hepatic tissue [48,49,50,64], indicating that the therapeutic efficacy of PP2A inhibitors depends on precise cancer-targeted delivery systems. We have addressed this challenge by developing bi-specific peptides composed of a TPP and an IP module. Peptide drug conjugates are gaining importance in cancer therapy. For example, PEPAXTO^®^ (melphalan flufenamide), a peptide drug conjugate that enters cells by passive diffusion and releases the drug via the action of intracellular aminopeptidases, has been recently approved for clinical use. The advantage of our TPP-IPs over other peptide drug conjugates like PEPAXTO is their high specificity for malignant cells. The TPPs target receptors that are highly expressed in tumoral cells and, once inside the cell, they only dissociate the pool of PP2A associated to SET, without any effect on the free PP2A and SET partners.

Aberrant expression of SET has been reported in other cancers such as leukemia, breast cancer, and colon, liver, and lung carcinoma [65,66,67,68,69]. The oncogenic role of SET in HCC was first suggested by Fukukawa et al. [70] who demonstrated that SET expression is highly upregulated in progressive HCC, indicating that SET may be involved in HCC development. Furthermore, SET activity is associated with development of resistance to chemotherapies [71,72,73]. Results obtained with patient primary cells support the oncogenic role of SET in HCC, suggesting that SET may serve as a novel biomarker to guide treatment in patients with HCC [69].

In conclusion, we report a selective tumoral internalization and apoptotic effect of peptides with potential clinical applications in liver cancer. The correlation between TPP receptor expression levels, TPP-IP internalization levels, and tumor aggressiveness score suggests that TPP receptor expression could serve as a marker of HCC aggressiveness.

## Figures and Tables

**Table 1:** Clinical characteristics of the patients.

Patient	Sex	Age	Type Tumor	AFP	Log_10_ AFP	Partially Encapsulated (0/1)	Satellite Nodule (0/1)	Vascular Invasion (0/1)	Differentiation (1/2/3) ^1^	Macrotrabecular (0/1)	Aggressiveness ^2^	Aggression Class
1	F	48.5	Hepatocellular adenoma	1.6	0	0	0	0	0	0	0	Null
2	M	65.3	Necrotic lymph node	2.6	0	0	0	0	0	0	0	Null
3	F	53.5	Angiomyolipoma	4.9	0	0	0	0	0	0	0	Null
4	M	56.5	Microtrabecular and pseudoglandular, Nuclear grade 2	7.7	0	1	0	0	2	0	3	Moderate
5	F	47.4	Microtrabecular	1010	3	1	1	0	2	0	7	High
6	F	59.9	Trabecular, Edmondson grade 2, nuclear grade 2	28,000	4	1	0	0	2	0	7	High
7	M	76.4	Microtrabecular and pseudoglandular	6662	3	1	0	0	2	1	7	High
8	M	47.1	Macro-trabecular, Edmonson grade 3, nuclear grade 3	6	0	1	1	1	2	1	6	High
9	M	73.4	Edmondson grade 3, nuclear grade 3	1.4	0	1	0	0	2	1	4	Moderate
10	M	67.3	Edmonson grade 2, nuclear grade 2	6.4	0	1	0	0	2	0	3	Moderate
11	M	57.5	Macrotrabecular	5.1	0	1	1	1	2	1	6	High
12	M	69.3	Trabecular, Edmonson grade 2, nuclear grade 2	343	2	1	0	2	2	0	6	High
13	M	68.7	Edmonson grade 2 HCC, nuclear grade 2	2.5	0	1	0	2	2	0	3	Moderate
14	M	78.8	Trabecular	341	2	0	0	2	2	0	5	Moderate

^1^ Well differentiated HCC = 1, moderately differentiated HCC = 2, undifferentiated HCC = 3. ^2^ Sum of Log_10_ AFP, partially encapsulated, satellite nodule, vascular invasion, differentiation, macrotabecular HCC (min = 0, max: 7).

**Table 2:** Sequence of the peptides used in this study.

Peptide ID	Sequence
iRGD-IP	FITC -Ahx-ETVTLLVALKVRYRERIT-Ahx-CRGDKGPDC-CONH_2_ (C-C disulfide bond)
RPARPAR-IP	FITC -Ahx-ETVTLLVALKVRYRERIT-Ahx-RPARPAR-OH
LinTT1-IP	FITC -Ahx-ETVTLLVALKVRYRERIT-Ahx-AKRGARSTA-CONH_2_
TT1-IP	FITC -Ahx-ETVTLLVALKVRYRERIT-Ahx-CKRGARSTC-CONH_2_ (C-C disulfide bond)

Ahx: aminohexanoic acid.

**Table 3:** Immunohistochemical characteristics of the patients.

Patient	CK19	HepPar	GPC3	Nuclear β-Catenin	Glutamine Synthetase
1	−	+	−	+	−
2	necrosis	necrosis	necrosis	necrosis	necrosis
3	−	−	−	−	−
4	−	+	+	10–20%	+++
5	−	−	−	0	0
6	−	+	−	−	++
7	−	+++	−	+	+++
8	+	+++	−	−	−
9	−	+	+	−	−
10	−	+	−	−	+
11	−	+++	+	−	−
12	−	+++	+	−	−
13	−	+++	−	−	−
14	−	+	+++	+	−

0: no material was available.

## Data Availability

Data are available upon request.
